# Supplementary material for: Pain and satisfaction: the case of isolated COVID-19 patients of Pakistan
Source: PeerJ. 2021 Aug 5;9:e11859. doi: 10.7717/peerj.11859 (PMC8349515; doi:10.7717/peerj.11859)
Supplement: Supplemental Information 1 [file peerj-09-11859-s001.pdf]

Questionnaire for COVID-19 patients

The questionnaire is designed to collect the psycho-social aspects of COVID-19 positive patients isolation in hospitals. The anonymity of patients will be assured.

1. Gender
   - Male
   - Female
   - Other
2. Marital status:
   - Unmarried / Never Married
   - Currently Married
   - Widow/widower
   - Divorced
3. Age (in years):
4. SPOUSE'S Age (in years):
5. Level of education (encircle the option)
   1-Primary
   2-Secondary
   3-High School
   4-College
   5-Graduate
   6-Post-graduate

   5a. SPOUSE level of education:
   1-Primary
   2-Secondary
   3-High School
   4-College
   5-Graduate
   6-Post-graduate
6. Mother level of education (encircle the option)
   1-Primary
   2-Secondary
   3-High School
   4-College
   5-Graduate
   6-Post-graduate

   6a. Father level of education:
   1-Primary
   2-Secondary
   3-High School
   4-College
   5-Graduate
   6-Post-graduate
7. Employment status:
   1-Full time
   2-part time
   3-Unemployed
   4-Full time student
   5-Retired

   8a. Spouse's employment status:
   1-Full time
   2-part time
   3-Unemployed/Housewife
   4-Full time student
   5-Retired
8. Occupation:
9. SPOUSE'S occupation:…………………………………………………………………
10. Household's size:______
   The number of dependants -Adults: ………… -Children: ………………………...
11. Adults or children for whom you are financially responsible for at least 50% of their support?

-No

-If YES, How many: …………………………..

12. Do you currently own or rent your home:
    - OWN
    - RENT
    - OTHER (……………………………………………….)
13. Are you head of the house?
    - Yes
    - No
14. What is your income per month?

    ____

15. My total income is enough for me to meet my monthly living expenses.
    - Strongly disagree
    - Disagree
    - Neutral
    - Agree
    - Strongly agree
16. What do you feel that your income is sufficient to meet your expenditures during this lockdown?

    1-Not at all adequate

    2-Can meet necessities only

    3-Can afford some of the things

    4-Can afford everything

    5-Afford everything & still save money
17. Do you think that a relief package from the government could help you?

    Yes No Don't want relief package
18. Overall, how satisfied are you with your life nowadays after pandemic?
    - Very dissatisfied
    - Dissatisfied
    - Neither satisfied nor dissatisfied
    - Satisfied
    - Very satisfied

19. Overall, to what extent do you agree that the things you do in your life are worthwhile?
    - Strongly disagree
    - Disagree
    - Neutral
    - Agree
    - Strongly agree

20. Overall, how happy did you feel about your life before pandemic?
    - Not very happy
    - Not happy
    - Neutral

- Happy
- Very happy

21. Are you satisfied with your economic situation during this lockdown where you are suffering from the disease as well?
    - Strongly disagree
    - Disagree
    - Neutral
    - Agree
    - Strongly agree

Fear of COVID-19

	**Questions**	**Strongly agree**	**Agree**	**Neutral**	**Disagree**	**Strongly disagree**
22	I am most afraid of coronavirus.					
23	It makes me uncomfortable to think about coronavirus-19					
24	My hands become clammy when I think about coronavirus-19					
25	I am afraid of losing my life because of coronavirus-19					
26	When watching news and stories about coronavirus-19 on social media, I become nervous or anxious					
27	My heart races or palpitates when I think that I have coronavirus-19					
28	Do you fear leaving the house?					

29. What is your pain level?
    - No pain
    - Mild
    - Moderate
    - Intense
    - Unspeakable

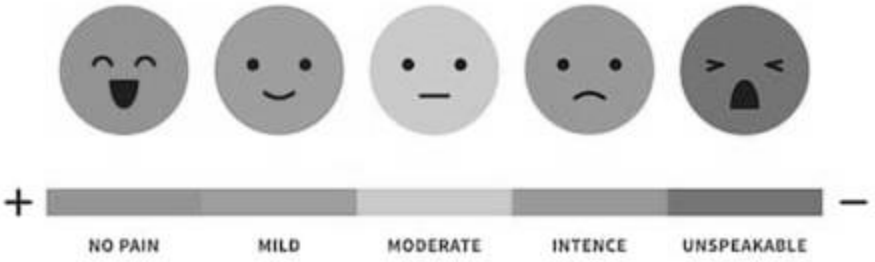

	Questions	Very dissatisfied	Dissatisfied	Neither dissatisfied nor satisfied	Satisfied	Very satisfied
30	Are you satisfied with the overall facilities in the isolation centre?					
31	Are you satisfied with the food facilities in the isolation centre?					
32	Are you satisfied with the timely provision of medicine in the isolation centre?					
33	Are you satisfied with the behaviour of peer patients with you?					
34	How much are you satisfied with the cleanliness of the isolation facility?					
35	How much are you satisfied with the behaviour of doctors?					
36	How much are you satisfied with the behaviour of nurses?					

37. Do you think that you got corona due to your negligence in prevention?

- Strongly disagree
- Disagree
- Neutral
- Agree
- Strongly agree

How much cost did you incur for the treatment of COVID-19 approximately?

___
