# Supplementary material for: Pain and satisfaction: the case of isolated COVID-19 patients of Pakistan
Source: PeerJ. 2021 Aug 5;9:e11859. doi: 10.7717/peerj.11859 (PMC8349515; doi:10.7717/peerj.11859)
Supplement: Supplemental Information 3 [file peerj-09-11859-s003.pdf]

Codebook for the raw data

The question numbers are same in the questionnaire as well as in the raw data.

Q1.

1=Female, 2=Male, 3=Others

Q2.

1=Unmarried/never married, 2=Currently married, 3=Widow, 4=Divorced

Q5, 5a, 6, 6a

1=Primary, 2=Secondary, 3=High school, 4=College, 5=Graduate, 6=Postgraduate

Q7, 8a

1=Full time, 2=Part time, 3=Unemployed, 4=Full time student, 5=Retired

Q11-Household size

No=0, Yes then mention the number

Q11a-Dependent adult

No=0, Yes then mention the number

Q11b-Dependent children

No=0, Yes then mention the number

Q12

1=Own, 2=Rent, 3=Other

Q13

1=Yes, 2=No

Q15

1=Strongly disagree, 2=Disagree, 3=Neutral, 4=Agree, 5=Strongly agree

Q16

1=Not at all adequate, 2=can meet necessities only, 3=can afford some of the things, 4=can afford everything, 5=afford everything and still save money

Q17

1=Yes, 2=No, 3=Don't want relief package

Q18

1=Very dissatisfied, 2=Dissatisfied, 3=Neither satisfied nor dissatisfied, 4=Satisfied

Q19

1=Strongly disagree, 2=Disagree, 3=Neutral, 4=Agree, 5=Strongly agree

Q20

1=Not very happy, 2=Not happy, 3=Neutral, 4=Happy, 5=Very happy

Q21

1=Strongly disagree, 2=Disagree, 3=Neutral, 4=Agree, 5=Strongly agree

Q22-28

1=Strongly agree, Agree, Neutral, Disagree, Strongly disagree

Q29

1=No pain, 2=Mild, 3=Moderate, 4=Intense, 5=Unspeakable

Q30-36

1=Very dissatisfied, 2=Dissatisfied, 3=Neither satisfied nor dissatisfied, 4=Satisfied

Q37

1=Strongly disagree, 2=Disagree, 3=Neutral, 4=Agree, 5=Strongly agree
